# Editorial: ICF in teaching, training and education - retrospective for future concepts - what remains of 20 years of ICF in education?

**DOI:** 10.3389/fresc.2025.1559098

**Published:** 2025-02-13

**Authors:** Sandra Kus, Andrea Glässel, Anita Kidritsch

**Affiliations:** ^1^ Chair of Public Health and Health Services Research, Institute of Medical Information Processing, Biometry and Epidemiology (IBE), Faculty of Medicine, LMU Munich, Munich, Germany; ^2^ Pettenkofer School of Public Health, Munich, Germany; ^3^ Institute of Biomedical Ethics and History of Medicine, Faculty of Medicine, University of Zurich, Zurich, Switzerland; ^4^ Institute of Public Health, School of Health Sciences, ZHAW, Winterthur, Switzerland; ^5^ Institute of Health Sciences, St. Pölten University of Applied Sciences, St. Pölten, Austria

**Keywords:** ICF (International Classification of Functioning, Disability and Health), functioning, person-centred, health professionals, rehabilitation, training, interprofessional collaboration, interprofessional education

**Editorial on the Research Topic**
ICF in teaching, training and education - retrospective for future concepts - what remains Of 20 years of ICF in education?

To align primary health care or rehabilitation services with people's needs and capabilities, it is critical that users can communicate their functioning to social and medical services and moreover, better inform health policy. The World Health Organization's International Classification of Functioning, Disability, and Health (ICF) provides a common language for describing functioning. Since the ICF's adoption in 2001, efforts have been made to educate and train people on using the ICF. Ongoing ICF education and training in different settings is essential for the classification's dissemination and use as a global standard. In the meanwhile, valuable resources for high-quality learning and teaching materials are provided in different languages.

This Research Topic consists of six articles, each describing experiences of educating, teaching and training ICF in academia and practical field teaching. A common theme emerges - while the ICF is being incorporated into bachelor's degree programs for various health professions, preparing students for applying the framework in real-world clinical settings remains challenging. Rehabilitation centres report needing to develop their own practical training materials to bridge this gap.

To address this issue, greater collaboration is needed between higher education institutions and clinical practice settings. By working together to develop case-based, hands-on training approaches, we can better equip the next generation of healthcare professionals with the skills and confidence to utilize the ICF framework. Innovative teaching methods, such as interprofessional group work and problem-based learning, show promise in facilitating this transfer of knowledge. Evaluations of in-person workshops demonstrate the value that healthcare providers place on this type of practical, applied learning.

## How to teach ICF best and what practices are established in between?

Kirschneck et al., describe the evaluation of an in-person interactive ICF training in Germany, which assessed workshop organization and knowledge gained through Likert scale and open-ended questions. Between 2017 and 2020, LMU Munich conducted 12 trainings with 191 participants. Of 151 respondents (79.1% response rate), most were from the social (50.3%), clinical (23.8%), and administrative (20.5%) sectors. 42.4% found the content highly relevant, and 82.1% would recommend the training. Suggestions for improvement focused on content, including themes on children, youth, and integration assistance.

Based on findings from three projects, Simon et al., demonstrate how the biopsychosocial model of ICF can serve as a common language between different health professions and to unify understanding of health and functioning. Their article outlines innovative educational approaches which used the ICF to facilitate interprofessional discussions, participatory decision-making, and a more holistic, person-centred approach to team-based, coordinated care. However, the authors also note that a shift in professional culture and the development of interprofessional competencies are required.

## What concepts, modules, and formats for ICF teaching have been developed?

The teaching example from Glässel & Hippold, describes a pilot study exploring the ICF model's integration into an interprofessional elective for bachelor students from healthcare professions, emphasizing patient-centred care. Using narratives from the DIPEx (Data Database of Patients Experiences), students collaboratively analysed real patient experiences, fostering reflection on biopsychosocial perspectives. Through group discussions, structured reflections, and presentations, students deepened their understanding of the ICF model as a practical tool and gained insights into patient-centred interprofessional collaboration. Findings highlight the value of authentic patient experiences in enhancing empathy, communication, and teamwork among future healthcare professionals.

To implement the ICF in neurorehabilitation, essential skills must be integrated into healthcare professionals' basic training. Aftenberger & Taxer, present a concept, developed at the Institute of Physiotherapy at FH JOANNEUM in Graz, to teach these skills in a structured way, linking the ICF with the Clinical Reasoning Model (CR). Competencies are built over six semesters, with a focus on neurorehabilitation in later years. Interprofessional group work and problem-based courses support skill transfer. This article explores how the ICF can be integrated into bachelor's degree programs for physiotherapy and other healthcare programs, sharing experiences and best practices.

## What has been achieved with ICF and how can this succeed in the future? What tools are available for transferring knowledge about ICF and how are they evaluated?

Higher education institutions (HEIs) provide ICF education, but it may not fully prepare graduates to implement it in clinical settings. As experienced by Paltamaa et al., the education offered by HEIs often doesn't align with clinical practice needs, possibly due to gaps in training students versus professionals in rehabilitation. The article discusses the need for ICF training in practice and strategies to address it. Through the Erasmus + INPRO project, 18 ICF-based materials were developed for rehabilitation centres to promote person-centred, interprofessional practice. The authors emphasise the need for further collaboration between HEIs and clinical settings to enhance and expand these resources.

South Africa struggles with the effects of problematic substance use. The Community Oriented Substance Use Program (COSUP) described by Van Rensburg et al., is a harm-reduction initiative using the ICF framework to assess client functioning. A cross-sectional study in January 2023 used the COSUP Client Functioning Tool, with 23 Likert-scale questions and open-ended feedback. The results show most clients are unemployed working-age African males. While they cope physically, they need more mental health support. Key concerns include stress, anxiety, boredom, and lack of support. Despite challenges, some clients express hope, highlighting the program's positive impact.

As the articles in this Research Topic primarily focused on ICF education and training, it will be important for future research to improve the integration and implementation of the ICF framework in education and clinical practice, to enhance interprofessional education and collaboration in healthcare programs, as well as to develop interactive practical, case-based learning approaches based on patient narratives to teach the ICF and enable its application. By aligning education, training, and real-world application, we can bridge the gap and realize the full potential of the ICF framework to transform healthcare delivery and improve patient outcomes. The time is now to make this a reality.

A big thank you to all the authors and ICF enthusiasts who have remained faithful to ICF development and research over the years and have also incorporated it into the teaching formats.

